# Thermostable Proteases from *Geobacillus*: Production, Characterization, Structural Stability Mechanisms and Biotechnological Applications

**DOI:** 10.3390/microorganisms13112455

**Published:** 2025-10-27

**Authors:** Meng Wang, Jun-Wei Wang, Jun-Hui Cheng

**Affiliations:** 1 Qingdao Hospital, University of Health and Rehabilitation Sciences (Qingdao Municipal Hospital), Qingdao 266071, China; 2 School of Health and Life Sciences, University of Health and Rehabilitation Sciences, Qingdao 266113, China; 3 Institute of Biochemical Engineering, College of Materials Science and Engineering, Qingdao University, Qingdao 266071, China

**Keywords:** thermostable proteases, *Geobacillus*, characterization, structural stability, biotechnological applications

## Abstract

Proteases play key roles in many industrial processes and account for the majority of global enzyme sales. *Geobacillus* isolates from extreme environments such as marine hydrothermal vents are capable of producing high yields of proteases with thermophilic properties. Many proteases produced by *Geobacillus* species have been extensively studied, some of which have been purified and characterized. In addition, the high thermal stability largely depends on structural stability. Based on X-ray crystallography, several factors have been found to affect the structural stability of the thermostable proteases of *Geobacillus*. Moreover, the thermostable proteases of *Geobacillus* have a wide range of biotechnological applications, such as in detergent, food, bioremediation, leather-processing and textile industries. Therefore, this review focusses on the thermostable proteases of *Geobacillus*, including their characteristics, structural stability mechanisms and biotechnological applications. It will help the development of utilizing thermostable protease resources and enhancing their suitability for use in various industrial applications.

## 1. Introduction

Proteases, also known as peptidases or proteinases, are enzymes that catalyze the breakdown of proteins into short peptides or free amino acids by hydrolyzing peptide bonds [1]. Based on the role of amino acids in the active site, proteases are grouped into different types in the latest release (release 12.5) of the MEROPS database (https://www.ebi.ac.uk/merops, accessed on 9 October 2025): aspartic (A) protease, cysteine (C) protease, glutamic (G) protease, metallo (M) protease, asparagine (N) protease, mixed (P) protease, serine (S) protease, threonine (T) protease and unknown (U) protease [2]. Proteases have been determined to play crucial parts in the degradation of organic nitrogen compounds [3,4]. Proteases can be categorized into low-temperature, medium-temperature, and thermophilic proteases based on their optimal temperature.

Generally, thermophilic proteases have a maximum activity at high temperatures or exhibit sustained high activity at high temperatures for extended periods. Up to now, numerous thermophilic proteases have been produced, purified and characterized from thermophiles, which can thrive in temperatures above 50 °C [5]. Compared to mesophilic proteases, thermophilic proteases exhibit faster biological reactions, lower viscosity, and reduced susceptibility to contamination under high-temperature conditions [6]. The advantages have sparked curiosity about the extracellular protease activities of thermostable bacteria, making the thermostable proteases attractive to the biotechnology industries such as the detergent industry, food industry, and sludge biodegradation [7,8,9]. The most typical thermostable protease is thermolysin produced by *Bacillus thermoproteolyticus*, which has been utilized in the production of the artificial sweetener aspartame [10].

As the major sources of thermoactive proteases, members of the genus *Geobacillus* were frequently isolated and extensively studied during the last decades. *Geobacillus* was first proposed in 2001, which was transferred from many thermophilic members of the genus *Bacillus* on the basis of 16S rRNA gene sequence analysis [11]. This original genus description included six species: *G. kaustophilus*, *G. thermocatenulatus*, *G. thermodenitrificans*, *G. thermoleovorans*, *G. thermoglucosidasius* and the type species *G. stearothermophilus*. Subsequently, nine additional species, *G. caldoxylosilyticus*, *G. galactosidasius*, *G. icigianus*, *G. jurassicus*, *G. lituanicus*, *G. thermantarcticus*, *G. toebii*, *G. uzenensis*, and *G. vulcani*, have been validly described or transferred to the genus *Geobacillus*. More recently, *Geobacillus* has been re-assessed and sixty-three *Geobacillus* strains for which genome sequences are publicly available have been estimated for phylogenetic relatedness by whole genome approaches [12]. So far, 23 species of *Geobacillus* have been validly published (https://lpsn.dsmz.de/genus/geobacillus, accessed on 9 October 2025). The majority of *Geobacillus* strains grow in the temperature range 35–75 °C, with the optimum at 55–65 °C [13]. Hence, *Geobacillus* cultures have been isolated from diverse high temperature environments [14], such as compost [15], sewage sludge [16], hot springs [17,18,19,20], hot oil reservoir [21], natural gas wells [22], and geothermal effluents [23]. Notably, marine-derived *Geobacillus* strains have attracted increasing interest due to their unique adaptations to high salinity and pressure besides extreme temperatures. These microorganisms are widely distributed in shallow marine vents [24,25], and deep-sea hydrothermal vents [24,25,26,27,28]. They contribute to organic matter degradation and participate in biogeochemical cycling through the secretion of specific enzymes such as proteases, lipases, glucosidase, and amylase, which exhibit exceptional stability under extreme marine environments. *G. thermodenitrificans* strain V3 from the marine vents of the Aeolian Islands have been confirmed to degrade gelatin and casein at 60 °C, indicating its ability to produce thermophilic proteases [29]. Another protease-producing strain of *Geobacillus* isolated from undersea fumaroles, strain PLS A, has optimum activity at 60 °C [30]. A thermostable monoacylglycerol lipase from marine *Geobacillus* strain 12AMOR1 shows the highest hydrolysis activity at 60 °C, and the half-life was 60 min at 70 °C [31]. *Geobacillus* strain HTA-462 from a Mariana Trench sediment sample has been found to produce α-glucosidase, which shows optimal activity under alkaline (pH 9.0) and high-temperature (60 °C) conditions [32]. Two thermophilic bacteria from *Geobacillus* have been isolated from Likupang Marine Hydrothermal in Indonesia and they have an amylolytic index value at 55 °C [28]. The specific physiological properties make them promising candidates for marine biotechnology and extremophile enzyme applications.

To date, numerous thermophilic proteases from *Geobacillus* strains have been reported, mainly including enzymatic properties, structure and application potential. However, up to now, a holistic review of thermostable proteases of *Geobacillus* is still lacking. In this article, we summarize the studies on the thermostable proteases of *Geobacillus* species and industrial prospects of these proteases, by searching the literature with keywords ‘thermostable/thermophilic/thermoactive’, ‘protease/proteinase’ and ‘*Geobacillus*’ in the PubMed (https://pubmed.ncbi.nlm.nih.gov, accessed on 9 October 2025) and Web of Science (https://www.webofscience.com, accessed on 9 October 2025). This review covers the knowledge about characterization, structural stability mechanisms and biotechnological applications of thermostable proteases from *Geobacillus*. The review will help in making strategies for the exploitation of thermostable protease resources and improving their usability in different industrial applications.

## 2. Production, Purification and Characterization of Thermostable Proteases of *Geobacillus*

**Figure 1:**
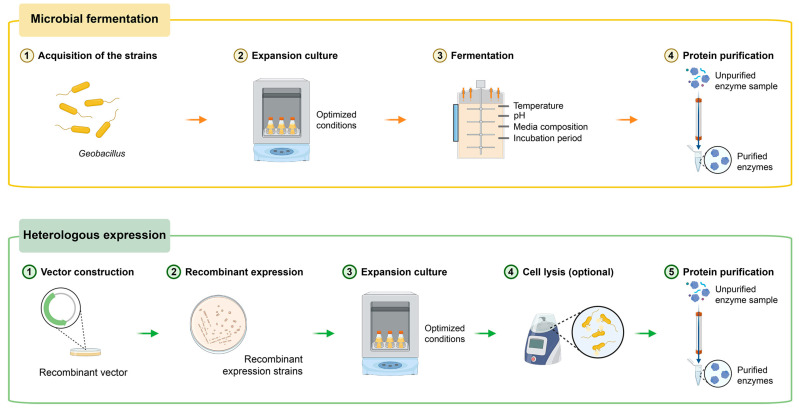
Two primary effective ways to produce thermostable proteases of *Geobacillus*.

Thermostable proteases unusually exhibit remarkable stability under high temperature conditions owing to the fact that organisms producing them are adapted to thermal environments. The genus *Geobacillus* is a typical thermostable group and most of them can grow above 50 °C. Consequently, they are considered to be excellent sources to produce thermophilic proteases. Currently, there are two primary effective ways to produce thermostable proteases of *Geobacillus* spp. (Figure 1).

(1) Microbial fermentation: This method involves the cultivation of *Geobacillus* spp. in bioreactors under controlled conditions to produce and secrete thermostable proteases. The proteases are then harvested, purified, and utilized. The thermophiles secreting thermostable proteases need to grow under optimum fermentation conditions, including optimum temperature, pH, media compositions and incubation period. For instance, the protease-production capacity of a *Geobacillus subterraneus* C2-1 isolate from a hot spring was investigated and optimized using a Plackett–Burman experimental design. The highest protease activity of the strain C2-1 was observed using 14.75 g/L glucose as the carbon source, 7.51 g/L yeast extract as the nitrogen source, an inoculum amount of 3.56%, a temperature of 56.4 °C, and a reaction time of 168 h [32]. The effects of various parameters on the protease-production capacity showed that *G. thermoglucosidasius* SKF4 had the highest ability to produce protease in 1% NaCl at 60 °C at pH 7 for 24 h. It showed a maximum capacity to utilize casein and yeast as sources of nitrogen and sucrose and fructose as good sources of carbon [33]. This process requires high temperature conditions and substantial energy for the cultivation of *Geobacillus* strains. The target thermophilic proteases are purified by ammonium sulfate precipitation and subsequent ion exchange chromatography [34,35].

(2) Heterologous expression: In this approach, the gene encoding the thermostable protease is isolated, cloned, and expressed in a suitable host organism, such as *Escherichia coli* or *Bacillus subtilis* [36,37,38,39]. By using recombinant DNA technology, researchers can produce large quantities of the thermostable protease in a mesophilic host organism, thereby conserving the energy for the cultivation of bacteria. After incubation, the recombinant cells are collected and disrupted by high pressure, and then the cell free crude enzyme extract is collected by centrifugation. For the recombinant proteases secreted outside the cell, the cell lysis step should be circumvented. Then the recombinant proteases are usually purified to homogeneity in a three-step procedure, including affinity chromatography, ion exchange chromatography and gel filtration chromatography [40].

The type of thermostable proteases of *Geobacillus* spp. is generally determined by measuring the enzyme activity after the addition of protease inhibitors such as PMSF (phenylmethylsulfonyl fluoride, an inhibitor of serine protease) and *o*-P (*o*-phenanthroline, an inhibitor of metalloprotease), a metal chelating agent such as EDTA (ethylene diamine tetraacetic acid) and EGTA (ethylene glycol tetraacetic acid), and some metal ions such as Ca^2+^, Mg^2+^, Fe^2+^, Zn^2+^. For instance, Zhu et al. [41] reported that the protease RH-1 of the *Geobacillus* strain YMTC 1049 was inhibited by 10 mM PMSF, which demonstrated that it belongs to the serine proteases family. Additionally, the keratinolytic proteinase RecGEOker derived from thermophilic bacterium *G. stearothermophilus* AD-11 was strongly inhibited by *o*-phenanthroline (29.5% residual activity), suggesting that RecGEOker is a kind of metalloproteinase [42].

Compared with mesophilic proteases, some thermophilic proteases isolated and characterized from thermophiles have advantages in thermostability. The biochemical characteristics of the thermostable proteases of *Geobacillus* spp. are summarized in Table 1. The studied thermostable proteases from *Geobacillus* mostly exhibited optimum activity at 55–70 °C and are stable at a wide range of pH values (6–10). In particular, the thermophilic protease from *Geobacillus toebii* strain LBT 77 is extremely stable and quite active up to 70 °C and pH 13, which indicates that the protease of the *G. toebii* strain LBT 77 is a thermostable alkaline protease. The protease can remain completely stable at 70 °C after 180 min of incubation, with a half-life of 70 min at 95 °C. These exceptional characteristics make it a promising candidate for various applications [43].

## 3. Structural Stability Mechanisms of Thermostable Proteases of *Geobacillus*

There are many factors that affect the thermal stability of enzymes, mainly including two aspects, intermolecular interaction and molecular conformation. Intermolecular interaction can make proteins fold more tightly and thus increase the rigidity. Common intermolecular interactions include ionic bond, hydrogen bond, hydrophobic interaction, and disulfide bond [49,50,51,52,53]. In addition, the conformation of the enzymes also plays an important role in thermal stability. The structural strength depends on the overall flexibility and rigidity of the enzyme, which are conducive to conformational stability.

**Figure 2:**
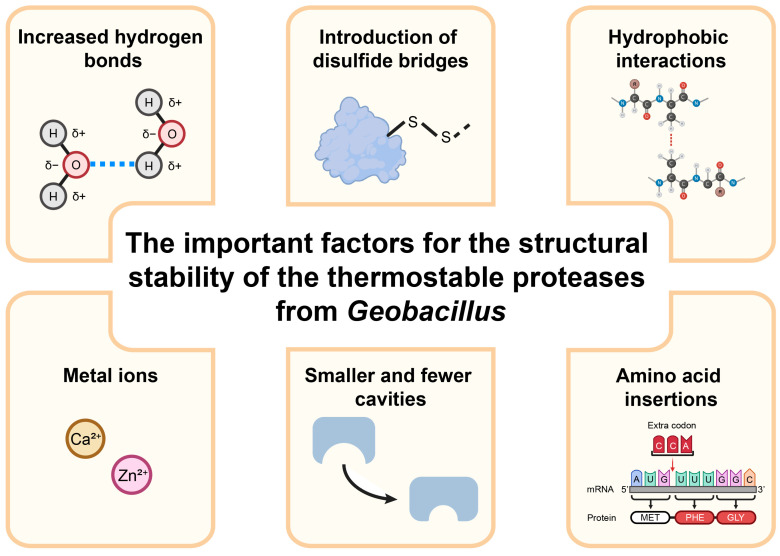
The important factors for the structural stability of the thermostable proteases of *Geobacillus*.

The proteases of *Geobacillus* spp. are usually characterized by high thermal stability and heat resistance, which largely depend on structural stability. X-ray crystallography has been utilized to analyze the structures of the thermostable proteases of *Geobacillus* [54,55]. Research findings have revealed that the thermostable proteases of *Geobacillus* have special secondary structures and amino acid sequences, facilitating their ability to sustain catalytic activity in high-temperature environments. There are several important factors for the structural stability of the thermostable proteases of *Geobacillus* spp. (Figure 2).

### 3.1. Increased Hydrogen Bonds

Hydrogen bonds play a crucial role in the thermal stability of enzymes, which not only occur between protein molecules, but also form interactions with adjacent water molecules. The formation of hydrogen bonds should meet the following two conditions: first, the distance between the hydrogen bond donor and the acceptor should be less than 3 Å; second, the angle of the hydrogen bond donor–hydrogen atom–hydrogen bond acceptor should be between 90° and 180° [56]. The number of charged neutral hydrogen bonds (i.e., between a side chain atom of a charged residue and either a main chain atom of any residue or a side chain atom of a neutral residue) has also been demonstrated to have a strong correlation with the protein thermostability [57]. Several studies have revealed that enhancing the thermostability of industrial enzymes can be achieved by promoting the formation of hydrogen bonds through single substitutions [58,59,60,61,62]. Additionally, the buried Ala-170 of the neutral proteinase of *G. stearothermophilus* was replaced by serine (Ser), and hence an extra high-quality Ser-170-OH-Asn-241-Oδ hydrogen bond was formed. Molecular dynamics simulations indicated that the increased hydrogen bond could enhance the thermostability of the protease. The inclusion of the hydroxy group of Ser-170, which only functions as a donor but not as an acceptor in the folded mutant enzyme, offsets the favorable effect of the hydrogen-bonding potential of Asn-241-Oδ [63].

### 3.2. Introduction of Disulfide Bridges

The utilization of disulfide bridges is an effective strategy to enhance the stability of the thermophilic proteins, which stabilizes proteins by decreasing the entropy of the unfolded structure, primarily through entropic effects [56]. The entropic effect of the disulfide bridge grows in direct proportion to the logarithm of the number of residues separating the two bridged cysteines. Therefore, increasing the disulfide bridges can decrease the entropy of the protein unfolding state, thereby stabilizing the conformation of enzymes. So far, more than one report has shown that unspecific proteases from *Geobacillus* spp. can be stabilized dramatically by the introduction of disulfide bridges. The thermolysin-like protease produced by *G. stearothermophilus* possesses a crucial surface area in the N-terminal domain that is essential for thermal stability. The introduction of a disulfide bond into the N-terminal domain by the double mutation G8C/N60C resulted in an extraordinarily thermostable enzyme with a half-life of 35.9 min at 92.5 °C, which was increased more than 120-fold, from less than 0.3 to 35.9 min [64,65].

### 3.3. Hydrophobic Interactions

The hydrophobic interactions could be a prominent element for the thermostability of proteins. It was proposed that hydrophobic forces play a crucial role in thermostability and act as the primary driving force for protein folding [66,67]. Hydrophobic interactions between non-polar amino acid residues such as tryptophan, methionine, valine, leucine, isoleucine, alanine, phenylalanine, proline, and glycine significantly contribute to the conformational stability of proteins [68,69]. During the folding process, the hydrophobic residues of the enzymes are obscured within the protein structure to produce hydrophobic effects conducive to protein stability, which helps to maintain the thermal stability of the enzymes. Pace et al. [70] found that each additional buried methyl group during protein folding contributes an average stability increment of 1.3 (±0.5) kcal/mol. By constructing three-dimensional models of wild-type Npr-ste and its mutants, it was deduced that the cavity-filling mutations in the hydrophobic core, which could improve hydrophobic packing and Van der Waals interactions in the protein interior, had positive effects on the thermostability of the protease from *G. stearothermophilus* [71].

### 3.4. Metal Ions

**Figure 3:**
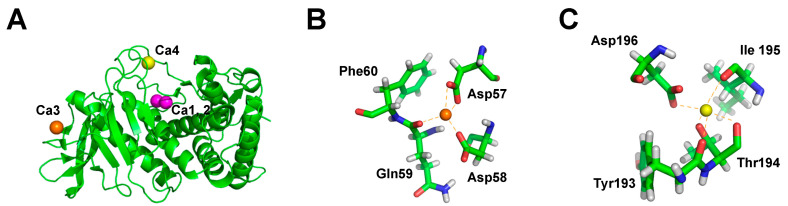
(**A**) The overall structure of the thermolysin-like protease from *Geobacillus stearothermophilus.* The double calcium-binding site (Ca1, 2) is shown as a magenta sphere and the single calcium-binding sites (Ca3 and Ca4) are shown as an orange and yellow sphere, respectively. (**B**) The atomic details of the calcium-binding site 3 (Ca3). (**C**) The atomic details of the calcium-binding site 4 (Ca4).

It has long been known that a reason for the stability of thermophilic proteases is the presence of metal ions (Ca^2+^, Zn^2+^) to enhance molecular stability. For instance, calcium ions have been considered to contribute to the stability of thermolysin produced by *G. stearothermophilus* and are also crucial for the stability of thermolysin-like protease (TLP) [72]. The TLP from *G. stearothermophilus* (TLP-ste) binds four calcium ions, including one double (Ca1, 2) and two single (Ca3, Ca4) calcium-binding sites (Figure 3A). Since Ca3 and Ca4 are typically lacking in thermolabile TLPs, it is likely that they are critical for determining the stability of TLPs. The atomic details of the Ca3 and Ca4 binding sites in TLP-ste are shown in Figure 3B and Figure 3C, respectively. The mutations of Ca3 and Ca4 indeed reduced the thermal stability of TLP-ste. The unfolding of the TLP-ste region containing the Ca3 site is essential for thermal inactivation. It is not caused by the lack of calcium from the Ca3 site, but rather by unfolding of a region of TLP-ste whose stability depends on the occupancy of the Ca3 site [73].

### 3.5. Smaller and Fewer Cavities

Cavities are an inherent feature of protein structures, with research indicating that the majority of protein cores possess these voids [71,74]. Such cavities typically exhibit fewer Van der Waals interactions compared to locations in densely packed regions, rendering them energetically unfavorable. Filling the cavities by hydrophobic amino acids with bulkier side chains is considered as a thermostability factor [75]. Consequently, the thermophilic proteins known for their high stability tend to display smaller and fewer cavities [56,76]. The cavities in the hydrophobic core of the neutral protease from *G. stearothermophilus* were analyzed using a three-dimensional model that was inferred from the crystal structure of thermolysin, the highly homologous neutral protease of *B. thermoproteolyticus*. Site-directed mutagenesis was used to fill some of these cavities, producing slight effects on the thermostability. Those substitutions involving the larger side chains tended to result in more significant enhancement in the protease thermostability [71]. Additionally, Eijsink et al. [63] noted that the hydroxy group of Ser could improve stability by filling an internal cavity and an increase of 0.7 ± 0.1 °C in stability was obtained after the buried Ala was replaced by Ser.

### 3.6. Amino Acid Substitutions/Insertions

Protein amino acid composition has long been thought to be correlated to its thermostability and the thermophilic and mesophilic proteins have been found to have significantly different amino acid distributions. Based on the genome sequences of eight mesophilic and seven thermophilic organisms, it has been shown that more hydrophobic and more aromatic residues are found in thermophilic proteins than in mesophilic proteins [77]. Therefore, increasing the proportion of hydrophobic amino acids such as isoleucine and proline contributes to the tight packing of hydrophobic cores and helps to enhance the thermostability [69,78,79]. When prolines were inserted at the second position of β-turns or the N caps of α-helices, the stability enhancement was most noticeable [80]. In addition, due to the large side chains, amino acids like arginine and tyrosine may be beneficial in short-range local interactions and in long-range interactions. The guanidium group in arginine which can form salt bridges also contributes to enhancing the thermostability [56]. Veltman et al. [81] have reported that an arginine-rich three-residue insertion in the TLP produced by *G. stearothermophilus* CU21 appeared to make an important contribution to the stability of the protease.

Indeed, protein stability is not attributed to a single factor, but is often the result of multiple factors working together. Heat stable proteases of *Geobacillus* bacteria have attracted much attention due to their special structural properties. The studies of their structures and thermal stabilization mechanisms can provide a theoretical basis for the development and utilization of the thermostable proteases in the application of various fields. In the future, we need to study their structures and thermal stabilization mechanisms, which can provide theoretical guidance for biotechnological applications of the thermostable proteases of *Geobacillus*.

## 4. Biotechnological Applications of Thermostable Proteases of *Geobacillus*

**Figure 4:**
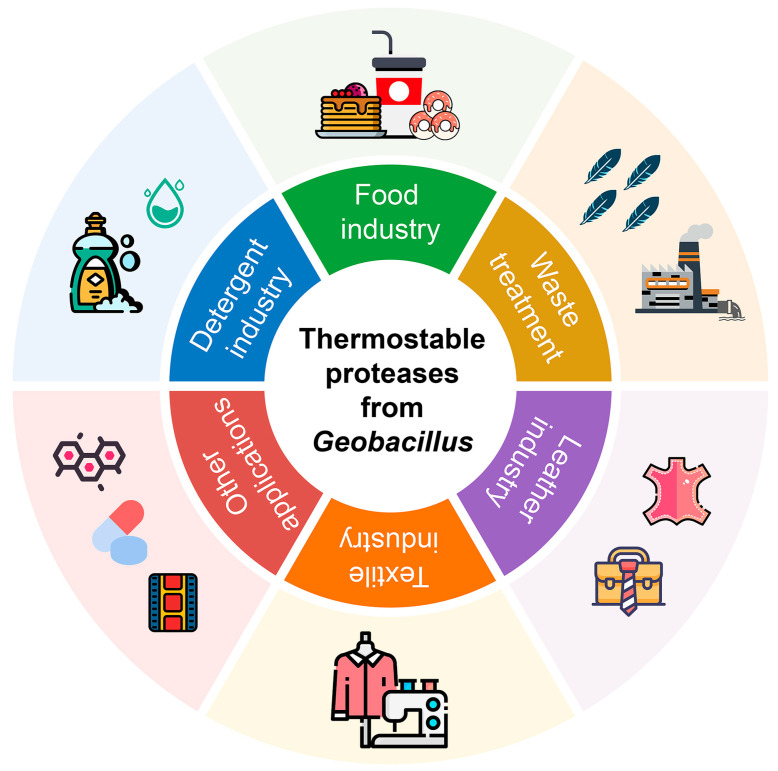
The biotechnological applications of the thermostable proteases of *Geobacillus*.

Common proteases exhibit optimal activity between 25 and 40 °C, which is not ideal for all industrial applications. Most industrial biotechnology processes are carried out in high temperature environments, which could cause the denaturation of mesophilic proteases. Due to the special structural and catalytic properties, the proteases of *Geobacillus* are capable of thermal tolerance. The major benefit of thermophilic proteases is that they can remain active at a high temperature for a long duration of time and using thermophilic proteases can reduce the losses during preparation, storage and industrial production. Therefore, the thermostable proteases are generally suitable for industrial biotechnology processing, which have attracted much attention, and they have found potential applications in detergent, food, bioremediation, leather processing and textile industries (Figure 4).

### 4.1. Detergent Industry

Heat-stable proteases are able to break down proteins, including grime and stains on clothing. They can maintain activities at high temperatures, so even when washing clothes at high temperatures, thermostable proteases can work well. Therefore, adding thermostable proteases to laundry detergents has the potential to significantly improve cleaning efficiency [82]. Bayoumi et al. [7] reported that the thermostable protease from *G. stearothermophilus* B78 could effectively remove a variety of stains such as blood, chocolate, apple, mango, strawberry and pomegranate. This was achieved through a 15 min treatment at 55 °C, using the protease alone or with Rabso detergent (an Egyptian detergent product). Similarly, another report [83] indicated that the protease derived from *G. stearothermophilus* successfully removed stains from a blood sample at 50 °C, exhibiting promising potential as a viable candidate for commercial use in detergents. A serine protease from *G. thermoglucosidasius* SKF4 was successfully used to remove blood stains from cloth at 80 °C when combined with detergent [48]. Moreover, because of the high thermostability and stability to surfactants and metal ions, the serine protease from *G.* strain GS53 might have a potential effect in the detergent industry [35].

### 4.2. Food Industry

For a considerable period, microbial proteases have played a significant role in the dairy, baking, and food-processing industries, including the production of some food additives [84]. The proteases produced by *Geobacillus* spp. have been extensively investigated for their applications in the food industry [85]. Thermolysin, a thermostable metalloendopeptidase produced by *G. stearothermophilus*, is used for the commercial synthesis of *N*-(benzyloxycarbonyl)-l-aspartyl-l-phenylalanine methyl ester, the precursor for the artificial sweetener aspartame [8]. The thermostable proteases are also involved in the hydrolysis of proteins for the preparation of hydrolysates which have bioactive potential, such as antioxidant, antidiabetic, and antihypertensive activities. The protein hydrolysates may be applied as nutraceuticals and functional food ingredients, potentially contributing to food quality and promoting human health [86]. Wu et al. [87] have reported that the protease secreted by *G. stearothermophilus* through high-temperature solid-state fermentation can improve the nutritional value and bioactivity of soybean meal. An alkaline serine protease from *G. stearothermophilus* CAU209 (GsProS8) displayed a good ability to hydrolyze whey protein at 50 °C to prepare antihypertensive hydrolysates. Therefore, it could be a potential candidate for the preparation of antihypertensive peptides. This property provided important insights for its applications in the food industry [88].

### 4.3. Waste Treatment

The thermostable proteases from *Geobacillus* have been used in the management of waste generated from various industries, such as the poultry industry. Several million tons of feather waste are generated worldwide by poultry-processing industries. The hydrolysis of insoluble feathers by the thermostable proteases into soluble peptides and amino acids is a cheaper and more effective way to produce valuable products which can be used as additives in fertilizers and feed. The keratinases (a type of protease) produced by *G. stearothermophilus* PTCC 1713 and *G. thermodenitrificans* PS41 could be used to degrade chicken feather at a high temperature. The introduction of these proteases secreted by *Geobacillus* in sustainable material development was a potential strategy for waste management, and has industrial applications in green technologies [89,90]. The thermostable keratinolytic protease RecGEOker from *G.* strain AD-11 is an effective biocatalyst for the environmentally friendly enzymatic biodegradation of substrates rich in keratin and high value hydrolysis products—small peptides obtained from keratin waste biodegradation could be suitable for industrial applications in white and green biotechnology [42]. Additionally, the thermophilic strains of *Geobacillus* are often used for sludge degradation by producing proteases. For example, the thermophilic protease was secreted by *G. stearothermophilus* TP-2, which resulted in a 23.2% reduction ratio in volatile suspended solids (VSS) after 12 h of sludge hydrolysis [9]. Inoculation with thermophilic strains (*G.* strain DX5, *G.* strain DX8, and *G.* strain DX11) at 65 °C reduced sludge VSS and the protease activity was 2584 U/L, which demonstrated that the protease of these thermophilic strains played important roles in the lysis of sewage sludge [91]. Similarly, the thermophilic bacterium *G. kaustophilus* X3 was shown to possess high relative protease activity, accelerating primary sludge hydrolysis [92,93]. *G. thermodenitrificans* DC8 screened from the compost could solubilize excess sludge to extract proteins for sludge reduction and resource recovery. Therefore, these works provide a potential strategy to realize high-efficient resource recovery in sludge waste treatment [94].

### 4.4. Leather-Processing Industry

Leather processing has traditionally depended on substantial amounts of water, alkali, and chemicals. This has led to the urgent requirement for process optimization and environmental pollution reduction in the leather manufacturing industry. Compared with the traditional method, the microbial proteases offer an environmentally friendly alternative [95]. The application of proteases can effectively enhance the water absorption in dehydrated skins, eliminate impurities and undesired proteins, and reduce the swelling of pelts. Furthermore, the proteases that possess the ability to break down elastin and keratin are helpful to generate high-quality leather with the desired attributes of strength, softness, cleanness and texture in a shorter period of time. The thermo-halostable protease produced by *G*. strain PLS A from undersea fumaroles has been reported to cause dehairing and produce soft leather, presenting a promising application in the leather-processing industry [38].

### 4.5. Textile Industry

Wool is a versatile natural fiber widely utilized in the textile industry, which is commonly used to make clothing, domestic textiles, and technical textiles. Wool is known for its capacity to absorb and expel moisture, which makes woolen clothing both warm and comfortable. Two-thirds of the wool fibers are utilized in the garment business, demonstrating the significance of the wool textile industry within the broader textile industry [96]. In the wool textile industry, wool processing involves oxidation of the wool surface by means of chlorination or softening agents. However, these chemicals are known to pose environmental hazards. To solve the problem, the proteases are implemented to reduce the felting propensity of wool and enhance the tactile qualities of fabrics by imparting a soft and smooth texture. The crude enzyme of the thermophilic *Geobacillus* strain has been successfully utilized to process wool fibers. The scanning electron microscope analysis revealed that the enzymatic treatment smoothed the outer layer of the wool fibers and improved the quality of the wool surface. Therefore, protease treatment is a promising eco-friendly alternative to chemical treatment in the wool textile industry, thereby aiding in the mitigation of environmental pollution [97].

### 4.6. Other Potential Applications

The thermostable proteases from *Geobacillus* are also used in clinical medicine and experimental reagents [8]. In addition, it was suggested that *G. stearothermophilus* protease could be used as a biocontrol agent due to its catalytic domains [30]. In addition, the thermostable proteases (Pz peptidases A and B) from *G. collagenovorans* MO-1 have been proved to contribute to collagen degradation [44]. Interestingly, the thermostable protease from *G. thermoglucosidasius* SKF has been used to hydrolyze the gelatin layer on X-ray film, facilitating the recovery of silver from X-ray film [48]. The thermostable protease produced by *G.* strain SBS-4S has been utilized as a supplement in poultry feed, and it has been shown to enhance weight gain, feed consumption and the feed conversion ratio in poultry [98,99]. In addition, many other thermostable proteases from *Geobacillus* with potential in various industries have been mined by genome and proteome analysis. For example, the proteases of *G. thermoleovorans* ARTRW1 with high melting point (*T*_m_) value have been found by thermal proteome profiling analysis, and they have significant industrial and biomedical prospects to accelerate thermophilic enzyme research and innovation [100].

Therefore, the thermostable proteases of *Geobacillus* spp. have numerous biotechnological applications, including in the detergent, food, bioremediation, leather-processing and textile industries. Their ability to maintain their activity at high temperatures makes them particularly useful in these applications. Further research on the properties and applications of these proteases is needed to fully exploit their potential.

## 5. Conclusions and Perspectives

Numerous proteases of *Geobacillus* spp. from high temperature environments, such as marine geothermal sites and hot springs, exhibit high activity, and the thermophilic property makes them meet industrial requirements. This review emphasizes the production, purification, characterization, structural stability mechanisms and industrial application of proteases from a number of *Geobacillus* strains. It has been found that the thermostable proteases of *Geobacillus* mostly exhibited optimum activity at 55–70 °C. There are several important factors for the structural stability of the thermostable proteases from *Geobacillus*, such as an increased hydrogen bond, introduction of disulfide bridges, hydrophobic interactions, metal ions, smaller cavities, amino acid substitutions/insertion and so on. Due to the thermophilic property and structural stability, the thermostable proteases of *Geobacillus* have biotechnological and industrial applications in detergent, food, bioremediation, leather-processing and textile industries. However, most thermophilic proteases of *Geobacillus* have not yet been commercially applied, and the obstacle lies in the difficulty of constructing their high-efficiency expression vectors.

Despite recent advancements, the current understanding of the thermostable proteases of *Geobacillus* remains incomplete, necessitating further investigation. In particular, three future research areas should be noted. First, more new thermophilic proteases from *Geobacillus* need to be mined. Many thermostable proteases from *Geobacillus* with potential in various industries have been found by genome and proteome analysis, but they need to be further heterologously expressed and purified. Second, the regulatory mechanism of *Geobacillus* expressing thermostable proteases urgently needs to be explored, which will be conducive to the construction of chassis cells with efficient expression. The new catalytic mechanism, and thermal stability mechanism of protease also needs to be explored. Third, efficient and scalable strategies are needed to improve the feasibility of the use of thermostable proteases from *Geobacillus* in industrial applications. The immobilization of enzymes and other technologies are proposed in the industrial applications of thermostable proteases of *Geobacillus*, to improve the utilization of protease and reduce application costs.

## Figures and Tables

**Table 1:** Characterization of thermostable proteases produced by *Geobacillus ^a^*.

Protease Source	Type of Protease	Optimum Temperature (°C)	Optimum pH	Thermal Stability	Application	Reference
*G. caldoproteolyticus* SF03	-	70–80	8.0–9.0	Retained 57% and 38% activity at 80 or 90 °C for 1 h	-	[16]
*G.* strain PA-9	-	70	6.5	-	-	[17]
*G.* strain PA-5	-	60	8.0	-	-	[17]
*G.* strain K13	-	65	8.0	-	-	[18]
*G.* strain PLS A	-	60	7	-	Biocontrol agent	[30]
*G.* strain GS53	Serine protease	50	8	Retained 85% activity at 85 °C for 6 h	Detergent industry	[35]
*G. stearothermophilus* B-1172	Serine protease	70	-	Retained 71% activity at 80 °C for 3 h	Biocontrol agent	[36]
*G. stearothermophilus*	-	65	7.5	Retained 80% activity at 65 °C for 1 h	Leather-processing	[38]
*G. kaustophilus*	Serine protease	55	8.5	-	Detergent industry	[39]
*G.* strain YMTC 1049	Serine protease	85	7.5	Stable at 65 °C for 10 h	-	[41]
*G. stearothermophilus* AD-11	Metalloproteinase	60	9	Retained higher than 50% activity at 70–80 °C for 2 h	Waste treatment	[42]
*G. toebii* LBT 77	Serine protease	95	13	Stable at 70 °C for 3 h	-	[43]
*G. collagenovorans* MO-1	Metallopeptidase	65	7.6	Half-life of 30 min at 70 °C	Collagen degradation	[44]
*G. collagenovorans* MO-1	Metallopeptidase	70	8.4	Half-life of 30 min at 75 °C	Collagen degradation	[44]
*G.* strain SBS-4S	Metalloprotease	70	7.5	-	-	[45]
*G.* strain SBS-4S	-	60	9.0	Stable at 60 °C for 2 h	Detergent industry	[46]
*G. stearothermophilus* TLS33	Metalloprotease	70	8.5	-	-	[47]
*G. stearothermophilus* TLS33	Metalloprotease	85	7.5	-	-	[47]
*G. stearothermophilus* TLS33	Metalloprotease	90	7.0	-	-	[47]
*G.* *thermoglucosidasius* SKF4	Serine protease	80	10	Half-life of 15 h at 80 °C	Detergent industry	[48]

*^a^*: The dashes represent that no data is available.

## Data Availability

No new data were created.
